# The Effect of Transverse Shear in Symmetric and Asymmetric End Notch Flexure Tests–Analytical and Numerical Modeling

**DOI:** 10.3390/ma13143046

**Published:** 2020-07-08

**Authors:** Konrad Dadej, Paolo Sebastiano Valvo, Jarosław Bieniaś

**Affiliations:** 1 Faculty of Mechanical Engineering, Lublin University of Technology, Nadbystrzycka 36, 20-618 Lublin, Poland; j.bienias@pollub.pl; 2 Department of Civil and Industrial Engineering, University of Pisa, Largo Lucio Lazzarino, I-56122 Pisa, Italy; p.valvo@ing.unipi.it

**Keywords:** asymmetrical end notch flexure, fracture toughness, laminates, delamination, transversal shear forces, compliance calibration, analytical modeling, finite element analysis

## Abstract

This paper focuses on the effects of transverse shear and root rotations in both symmetric and asymmetrical end-notched flexure (AENF) interlaminar fracture toughness tests. A theoretical model is developed, whereas the test specimen is subdivided into four regions joined by a rigid interface. The differential equations for the deflection and rotations of each region are solved within both the Euler–Bernoulli simple beam theory (SBT) and the more refined Timoshenko beam theory (TBT). A concise analytical equation is derived for the AENF deflection profile, compliance, and transverse shearing forces as a function of the specimen geometry, stacking sequence, delamination length, and fixture span. Modeling results are compared with numerical finite element analyses, obtaining a very good agreement. Performed analyses suggest that even in the case of symmetrical and unidirectional laminates considered as pure mode II fracture, a complex compression/tension and bending moment state is present, as well as a slight contribution of anti-planar shear at the vicinity of the crack tip.

## 1. Introduction

The end-notched flexure (ENF) test is a mechanical test in which a partially cracked beam specimen is loaded in a three-point bending configuration. The test was initially applied by Barrett and Foschi [1] for testing the mechanical properties of wooden beams. Afterwards, a theoretical model of the ENF specimen was proposed by Russell and Street [2], who determined the mode II interlaminar fracture toughness of graphite/epoxy composite specimens. The ENF test method has become popular to test both standard symmetric unidirectional laminates [3] and non-standard asymmetrical laminates [4], as well as multidirectional fibrous composite materials [5], adhesive joints [6], and fibre metal laminates [7,8].

Recently, the ENF test has become a standard method for the determination of mode II fracture toughness in unidirectional and symmetrical composite laminates [9]. The standard describes the procedure for the determination of the mode II interlaminar fracture toughness, based on the experimental compliance–calibration (CC) tests. Within these tests, the dependence between force and deflection of beam specimens is experimentally determined for a few different initial crack lengths. Typically, to obtain reasonable results, a three-point CC plot is sufficient [10]. To this aim, the compliances for 20 and 40 mm delamination lengths are obtained from loading–unloading tests within the elastic range of behavior, while the compliance for 30 mm is obtained from a fracture test. The ENF specimens in CC tests should be loaded to at least half of the critical force Pc for each crack length. For that reason, the appropriate CC tests have to be preceded by additional preliminary research to determine the estimated fracture toughness of the material and critical force for corresponding crack length. Subsequently, from the CC tests, the slope of the compliance versus cubed crack length is marked by the m parameter and described typically by the linear function determined by regression analysis. Finally, the critical strain energy release rate $G_{IIC}$ of the material can be obtained based on the already known m parameter, crack length, and maximum force $P_{max}$ (or some different criteria).

In this article, an analytical solution is presented for the specimen compliance as a function of geometry, stacking sequence, crack length, and applied downward force. The proposed method can be used for the virtual performing of CC tests, which can be applied for the theoretical verification of experimentally determined compliance–calibration line. 

The paper also addresses the influence of transverse shearing loads and resulting cross-section rotations on the compliance and internal forces of both symmetric ENF and asymmetric ENF (AENF) test specimens. The symmetric configuration (ENF) is characterized by equal thicknesses and stiffnesses of the top and bottom sublaminates; also, the delamination is placed in the neutral plane of the specimen. If there are exceptions to the typical ENF configuration, then the sample is called AENF, where the thickness and stiffness of the sublaminates differ, or delamination is not in the laminate’s neutral plane. Russel and Street [2] originally presented an analytical model of the ENF test based on Euler–Bernoulli simple beam theory (SBT). However, subsequent studies have proven that the application of the SBT model resulted in an underestimation of the energy release rate [11]. Carlsson et al. [12] presented an analysis of the ENF specimen based on the Timoshenko beam theory (TBT) [13], which on the contrary to the SBT, enables for the kinematic cross-section rotation of the beam, with respect to their neutral plane and resulted in increased beam compliance. As noted in a critical review by Valvo [14], in the literature, conflicting reports may be found addressing the effect of shear deformation on the fracture toughness of the laminates [15,16,17,18]. Anyway, shear deformation clearly influences the compliance of both symmetric and asymmetric ENF test specimens, whereas the energy release rate is influenced only for asymmetric specimens. 

This article presents a complete analytical solution for the deflection and compliance of AENF and AENF test specimens with arbitrary geometry and material and stacking sequence, crack length, and fixture span. For comparison, the analytical solutions are presented using both the SBT and the TBT. Analytical models are validated by performing numerical finite element analyses in ABAQUS software (2017, Dassault Systèmes Simulia Corp., Johnston, RI, USA), using a built-in model of lamina based on first-order shear deformation theory (FSDT) [19,20]. 

The equations linking the deflection of the AENF specimen with the applied downward force, laminate geometry, and actual crack length, have been derived, for standard symmetrical as well as asymmetrical multidirectional composites, bi-material beams, and more advanced fibre–metal laminates. Moreover, thanks to the proposed equations recorded in the mechanical testing central point, deflection of the specimen can be also used to estimate an actual crack length with an increased accuracy due to the shearing effect in TBT.

## 2. Problem Description

**Figure 1:**
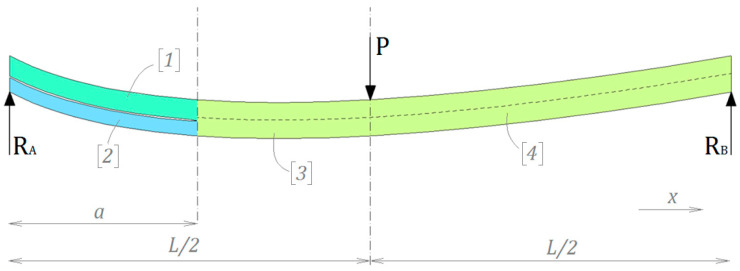
Theoretical division of the asymmetrical end-notched flexure (AENF) specimen.

The AENF test specimen can be divided into four regions—the cracked 1st and 2nd regions and non-cracked 3rd and 4th regions (respectively to the left and to the right of the central loading point), as shown in Figure 1. 

The starting point of the theoretical derivations is an assumption of equality of the reaction forces, $R_{A}$ and $R_{B}$, which counteract the load applied to the central point of the specimen. From the statics, the system is in equilibrium, when the sum of acting forces F and moments M is zero:

(1) $$\sum F=R_{A}-P+R_{B}=0\;\mathrm{and}\;\sum M=R_{B}L-\frac{1}{2}PL=0.$$

Then, the transverse shearing forces $Q_{1}$, $Q_{2}$, and $Q_{3}$ acting sublaminates to the left from centrum are positive:

(2) $$Q_{3}=Q_{1}+Q_{2}=\frac{1}{2}P$$

where the transverse shearing force $Q_{4}$ acting to the right from centrum are negative:

(3) $$Q_{4}=-Q_{3}=-\frac{1}{2}P.$$

Since the AENF specimen has an initial delamination, the shear and bending stiffnesses of the 1st and 2nd regions of a beam are different from those of the 3rd region. However, the latter are equal to those of the 4th region.

### 2.1. The Magnitude of Bending Moment Acting on Non-Cracked Part of the Beam

The overall transverse shearing force is constant between two loading points. Therefore, the actual value of the bending moment acting in the 3rd region of the AENF test specimen is a function of that shearing force and abscissa, x, along the beam length, which is equal to:

(4) $$M_{3}\left(x\right)=\frac{P}{2}x=Q_{3}x.$$

Thus, the bending moment in the 3rd part of the AENF specimen, on the cross-section adjacent to the 1st and 2nd regions of the AENF specimen, (at the crack tip) for x=a:

(5) $$M_{3}=\frac{P}{2}a=Q_{3}a.$$

The maximum bending moment acting of the specimen occurs for x=L/2:

(6) $$M_{max}=\frac{PL}{4}=\frac{Q_{3}L}{2}.$$

Finally, the bending moment acting in the 4th region of the AENF specimen can be described by the equation:

(7) $$M_{4}\left(x\right)=\frac{PL}{4}-\frac{P}{2}\left(x-\frac{L}{2}\right)=\frac{P}{2}\left(L-x\right)=Q_{3}\left(L-x\right).$$

**Figure 2:**
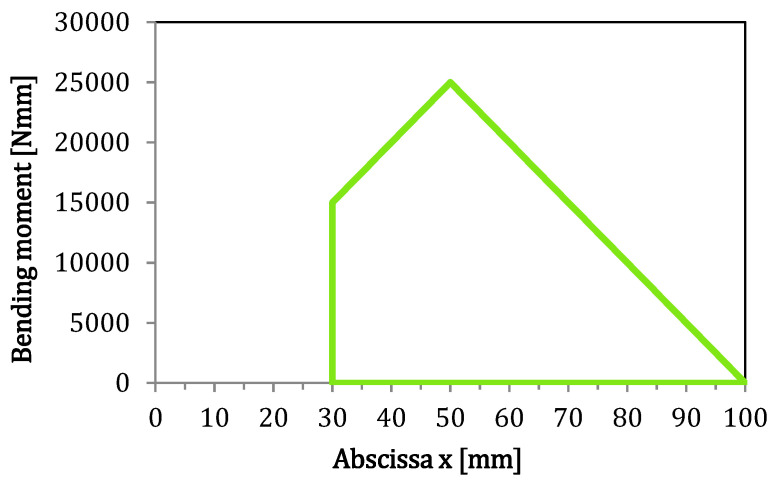
Bending moment diagram in the 3rd and 4th region of the AENF specimen.

Therefore, the bending moment diagram for the AENF specimen in the 3rd and 4th region is shown in Figure 2.

### 2.2. Shearing Forces and Bending Moment Acting on Delaminated Regions of the Specimen

**Figure 3:**
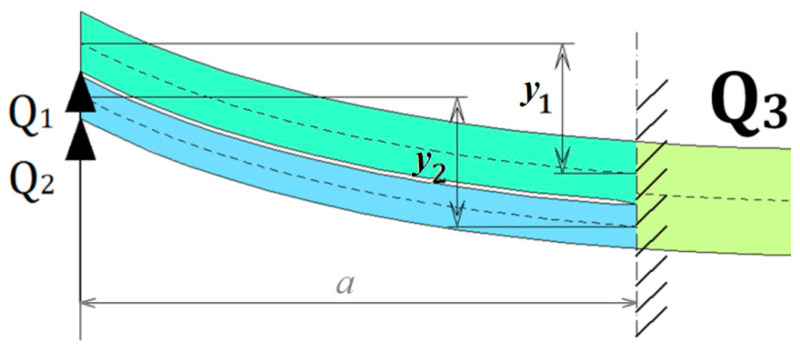
Assumption of equal deflection of sublaminate 1st and 2nd deflections during the AENF test.

As mentioned before, the delaminated part of the AENF specimen is divided into the 1st and 2nd regions. To derive the magnitude of shearing forces and bending moments acting on those regions, an assumption of equal deflections y of the 1st and 2nd regions is imposed, as shown in Figure 3. 

From that moment, the adopted theoretical model of the laminate has a significant impact on the further results of shearing forces. Thus, for further considerations, the model based on SBT, where the laminate cross-section subjected to bending remains perpendicular to the central plane, as well as more advanced model based on TBT, including cross-section rotation about central plane of the laminate.

## 3. Simple Beam Theory Solution

### 3.1. Deflection of Two Euler–Bernoulli Cantilever Beams Fixed at One End

The deflection of two Euler–Bernoulli’s cantilever beams $y_{\left[1\right]}^{SBT}$ and $y_{\left[2\right]}^{SBT}$ as a function of abscissa x can be written as:

(8) $$y_{\left[1\right]}^{SBT}\left(x\right)=\frac{Q_{1}x^{3}}{3E_{1}I_{1}}\;\mathrm{and}\;y_{\left[2\right]}^{SBT}\left(x\right)=\frac{Q_{2}x^{3}}{3E_{2}I_{2}}$$

where the longitudinal extensional stiffness $E_{1}$ and $E_{2}$ of the sublaminates 1 and 2 can be calculated according to Appendix A. The above regions of the beam have the same deflection ($y_{\left[1\right]}^{SBT}=y_{\left[2\right]}^{SBT}$), for specified abscissa x=a:

(9) $$\frac{Q_{1}a^{3}}{3E_{1}I_{1}}=\frac{Q_{2}a^{3}}{3E_{2}I_{2}}$$

which including Equation (2) into (9), gives:

(10) $$\frac{Q_{1}a^{3}}{3E_{1}I_{1}}=\frac{\left(Q_{3}-Q_{1}\right)a^{3}}{3E_{2}I_{2}}$$

after few simply calculations, the relation between shearing forces in sublaminates 1 and 3 are derived:

(11) $$Q_{1}=Q_{3}\frac{\chi^{SBT}}{1+\chi^{SBT}}\;\mathrm{and}\;Q_{2}=Q_{3}\left(1-\frac{\chi^{SBT}}{1+\chi^{SBT}}\right)$$

where:

(12) $$\chi^{SBT}=\frac{E_{1}I_{1}}{E_{2}I_{2}}.$$

**Figure 4:**
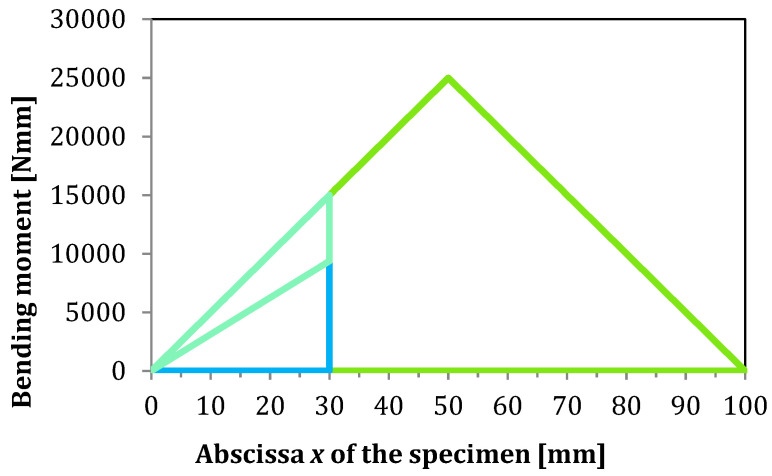
Bending moment diagram of the AENF specimen.

Therefore, the known value of shearing force $Q_{3}$ acting on the 3rd and 4th region may be used to determine shearing forces $Q_{1}$ and $Q_{2}$ acting on the 1st and 2nd regions, and further, for the equations of bending moments:

(13) $$M_{1}=Q_{1}x\;\mathrm{and}\;M_{2}=Q_{2}x$$

where the sum of shearing forces $Q_{1}$ and $Q_{2}$ must be equal to the shearing force $Q_{3}$ at the crack tip, as shown in Figure 4. Those shearing forces are differentiated by Young modulus and the moment of inertia of each region and must satisfy the equilibrium for the unite deflection of a whole structure.

### 3.2. Deflection Curve of the AENF Specimen

#### 3.2.1. First Integrations of the Differential Equations for Beams Curvatures

The defection $y_{\left[i\right]}$ of sublaminate *i*, for abscissa x is a function of the bending stiffness $E_{\left[i\right]}I_{\left[i\right]}$ and bending moment $M_{\left[i\right]}$ [13]:

(14) $$\frac{\partial^{2}y_{\left[i\right]}^{SBT}}{\partial x^{2}}E_{\left[i\right]}I_{\left[i\right]}=-M_{\left[i\right]}.$$

Four differential equations were proposed for four sublaminates:

(15) $$\frac{\partial^{2}y_{\left[1\right]}^{SBT}}{\partial x^{2}}E_{1}I_{1}=-Q_{1}x$$

(16) $$\frac{\partial^{2}y_{\left[2\right]}^{SBT}}{\partial x^{2}}E_{2}I_{2}=-Q_{2}x$$

(17) $$\frac{\partial^{2}y_{\left[3\right]}^{SBT}}{\partial x^{2}}E_{3}I_{3}=-Q_{3}x$$

(18) $$\frac{\partial^{2}y_{\left[4\right]}^{SBT}}{\partial x^{2}}E_{3}I_{3}=Q_{3}x-Q_{3}l.$$

According to the example shown in Timoshenko [13], the above-mentioned equations can be directly integrated to obtain first-order differential equations:

(19) $$E_{1}I_{1}\frac{\partial y_{\left[1\right]}^{SBT}}{\partial x}=\int E_{1}I_{1}\frac{\partial^{2}y_{\left[1\right]}^{SBT}}{\partial x^{2}}dx=-\int Q_{1}xdx=-\frac{Q_{1}x^{2}}{2}-c_{1}$$

(20) $$E_{2}I_{2}\frac{\partial y_{\left[2\right]}^{SBT}}{\partial x}=\int E_{2}I_{2}\frac{\partial^{2}y_{\left[2\right]}^{SBT}}{\partial x^{2}}dx=-\int Q_{2}xdx=-\frac{Q_{2}x^{2}}{2}-c_{2}$$

(21) $$E_{3}I_{3}\frac{\partial y_{\left[3\right]}^{SBT}}{\partial x}=\int E_{3}I_{3}\frac{\partial^{2}y_{\left[3\right]}^{SBT}}{\partial x^{2}}dx=-\int Q_{3}xdx=-\frac{Q_{3}x^{2}}{2}-c_{3}$$

(22) $$E_{3}I_{3}\frac{\partial y_{\left[4\right]}^{SBT}}{\partial x}=\int E_{3}I_{3}\frac{\partial^{2}y_{\left[4\right]}^{SBT}}{\partial x^{2}}dx=\int \left(Q_{3}x-Q_{3}l\right)dx=\frac{Q_{3}x^{2}}{2}-Q_{3}lx+c_{4}.$$

#### 3.2.2. First Boundary Conditions: Slopes of the Beams at Singular Points

The next step is to take into account the boundary conditions for the slopes of the three above functions in the crossing points. Thus, the slope of the beam on the 1st region is the same as the slope of the 3rd region at the point a, denoting the crack tip. Therefore, when the variable x is equal to crack length, a, the following equation is proposed:

(23) $$\frac{\partial y_{\left[1\right]}^{SBT}}{\partial x}\left(a\right)=\frac{\partial y_{\left[3\right]}^{SBT}}{\partial x}\left(a\right)$$

(24) $$-\frac{Q_{1}a^{2}}{2E_{1}I_{1}}-\frac{c_{1}}{E_{1}I_{1}}=-\frac{Q_{3}a^{2}}{2E_{3}I_{3}}-\frac{c_{3}}{E_{3}I_{3}}$$

which gives:

(25) $$c_{3}=\frac{E_{3}I_{3}Q_{1}a^{2}}{2E_{1}I_{1}}+\frac{E_{3}I_{3}c_{1}}{E_{1}I_{1}}-\frac{Q_{3}a^{2}}{2}.$$

Similarly, the slope of the 3rd region is equal to the 4th region in the central loading point of the ENF specimen. Therefore, the variable x is substituted by L/2:

(26) $$\frac{\partial y_{\left[3\right]}^{SBT}}{\partial x}\left(L/2\right)=\frac{\partial y_{\left[4\right]}^{SBT}}{\partial x}\left(L/2\right)$$

which is:

(27) $$-\frac{Q_{3}{\left(L/2\right)}^{2}}{2E_{3}I_{3}}-\frac{c_{3}}{E_{3}I_{3}}=\frac{Q_{3}{\left(L/2\right)}^{2}}{2E_{3}I_{3}}-\frac{Q_{3}l\left(L/2\right)}{E_{3}I_{3}}+\frac{c_{4}}{E_{3}I_{3}}$$

and after some calculation, that gives:

(28) $$c_{4}=-\frac{4c_{1}E_{3}I_{3}-L^{2}Q_{3}E_{1}I_{1}+2Q_{1}E_{3}I_{3}a^{2}-2Q_{3}E_{1}I_{1}a^{2}}{4E_{1}I_{1}}.$$

#### 3.2.3. Second Integration of the Beams Slope’s Equations.

Substituting integration constants $c_{3}$ and $c_{4}$ according to Equations (21) and (22), and then integrating, we get:

(29) $$y_{\left[1\right]}^{SBT}\left(x\right)=\int \frac{\partial y_{\left[1\right]}^{SBT}}{\partial x}dx=\int \left(-\frac{Q_{1}x^{2}}{2E_{1}I_{1}}-\frac{c_{1}}{E_{1}I_{1}}\right)dx=-\frac{Q_{1}x^{3}+6c_{1}x}{6E_{1}I_{1}}+d_{1}$$

(30) $$y_{\left[2\right]}^{SBT}\left(x\right)=\int \frac{\partial y_{\left[2\right]}^{SBT}}{\partial x}dx=\int \left(-\frac{Q_{2}x^{2}}{2E_{2}I_{2}}-\frac{c_{2}}{E_{2}I_{2}}\right)dx=-\frac{Q_{2}x^{3}+6c_{2}x}{6E_{2}I_{2}}+d_{2}$$

(31) $$\begin{array}{ll} & y_{\left[3\right]}^{SBT}\left(x\right)=\int \frac{\partial y_{\left[3\right]}^{SBT}}{\partial x}dx=\int \left(-\frac{Q_{3}x^{2}}{2E_{3}I_{3}}-\frac{c_{3}}{E_{3}I_{3}}\right)dx\\ = & \int \left(-\frac{Q_{3}x^{2}}{2E_{3}I_{3}}-\frac{\frac{E_{3}I_{3}Q_{1}a^{2}}{2E_{1}I_{1}}+\frac{E_{3}I_{3}c_{1}}{E_{1}I_{1}}-\frac{Q_{3}a^{2}}{2}}{E_{3}I_{3}}\right)dx\\ = & \frac{3Q_{3}a^{2}x-Q_{3}x^{3}}{6E_{3}I_{3}}-\frac{3Q_{1}E_{3}I_{3}a^{2}x+6c_{1}E_{3}I_{3}x}{6E_{1}I_{1}E_{3}I_{3}}+d_{3} \end{array}$$

(32) $$y_{\left[4\right]}^{SBT}\left(x\right)=\int \frac{\partial y_{\left[4\right]}^{SBT}}{\partial x}dx=\int \left(\frac{Q_{3}x^{2}}{2}-Q_{3}lx+c_{4}\right)dx=\frac{3Q_{3}L^{2}x-6Q_{3}Lx^{2}+6Q_{3}a^{2}x+2Q_{3}x^{3}}{12E_{3}I_{3}}-\;\frac{6Q_{1}E_{3}I_{3}a^{2}x+12c_{1}E_{3}I_{3}x}{12E_{1}I_{1}E_{3}I_{3}}+d_{4}.$$

#### 3.2.4. Second Boundary Conditions: Beam Deflections at Specimen Ends

The deflection at beam ends in the AENF test are zero, which enables specifying the following boundary conditions, for x=0:

(33) $$y_{\left[1\right]}^{SBT}\left(0\right)=-\frac{Q_{1}{\left(0\right)}^{3}+6c_{1}\left(0\right)}{6E_{1}I_{1}}+d_{1}=0$$

which gives:

(34) $$d_{1}=0$$

Similarly, for second beam end, for x=L:

(35) $$y_{\left[4\right]}^{SBT}\left(L\right)=\frac{3Q_{3}L^{3}-6Q_{3}L^{3}+6Q_{3}a^{2}L+2Q_{3}L^{3}}{12E_{3}I_{3}}-\frac{6Q_{1}E_{3}I_{3}a^{2}L+12c_{1}E_{3}I_{3}L}{12E_{1}I_{1}E_{3}I_{3}}+d_{4}=0$$

which gives:

(36) $$d_{4}=\frac{Q_{3}L^{3}-6Q_{3}a^{2}L}{12E_{3}I_{3}}+\frac{6Q_{1}E_{3}I_{3}a^{2}L+12c_{1}E_{3}I_{3}L}{12E_{1}I_{1}E_{3}I_{3}}.$$

#### 3.2.5. Third Boundary Conditions: Beam Deflections at Singular Points

Finally, the deflection curve of the 1st and 2nd regions at length a must be equal, which gives the following equation:

(37) $$y_{\left[1\right]}^{SBT}\left(a\right)=y_{\left[3\right]}^{SBT}\left(a\right)$$

(38) $$-\frac{Q_{1}a^{3}+6c_{1}a}{6E_{1}I_{1}}+d_{1}=\frac{3Q_{3}a^{3}-Q_{3}a^{3}}{6E_{3}I_{3}}-\frac{3Q_{1}E_{3}I_{3}a^{3}+6c_{1}E_{3}I_{3}a}{6E_{1}I_{1}E_{3}I_{3}}+d_{3}$$

which gives:

(39) $$d_{3}=\frac{3Q_{1}E_{3}I_{3}a^{3}+6c_{1}E_{3}I_{3}a}{6E_{1}I_{1}E_{3}I_{3}}-\frac{Q_{1}a^{3}+6c_{1}a}{6E_{1}I_{1}}-\frac{Q_{3}a^{3}}{3E_{3}I_{3}}=\frac{Q_{1}E_{3}I_{3}a^{3}-Q_{3}E_{1}I_{1}a^{3}}{3E_{1}I_{1}E_{3}I_{3}}.$$

Similarly, deflection of the 3rd and 4th regions at the half length of the span L/2 must be equal:

(40) $$y_{\left[3\right]}^{SBT}\left(L/2\right)=y_{\left[4\right]}^{SBT}\left(L/2\right)$$

which is:

(41) $$\begin{array}{l} \frac{3Q_{3}a^{2}\left(L/2\right)-Q_{3}{\left(L/2\right)}^{3}}{6E_{3}I_{3}}-\frac{3Q_{1}E_{3}I_{3}a^{2}\left(L/2\right)+6c_{1}E_{3}I_{3}\left(L/2\right)}{6E_{1}I_{1}E_{3}I_{3}}+\frac{Q_{1}E_{3}I_{3}a^{3}-Q_{3}E_{1}I_{1}a^{3}}{3E_{1}I_{1}E_{3}I_{3}}\\ =\frac{3Q_{3}L^{2}\left(L/2\right)-6Q_{3}L{\left(L/2\right)}^{2}+6Q_{3}a^{2}\left(L/2\right)+2Q_{3}{\left(L/2\right)}^{3}}{12E_{3}I_{3}}\\ -\frac{6Q_{1}S_{3}a^{2}\left(L/2\right)+12c_{1}E_{3}I_{3}\left(L/2\right)}{12E_{1}I_{1}E_{3}I_{3}}+\frac{Q_{3}L^{3}-6Q_{3}a^{2}L}{12E_{3}I_{3}}+\frac{6Q_{1}S_{3}a^{2}L+12c_{1}E_{3}I_{3}L}{12E_{1}I_{1}E_{3}I_{3}} \end{array}$$

which after some calculations, gives the simplest form of integration constant $c_{1}$, as a function of material constants and specimen geometry:

(42) $$c_{1}=-\frac{3L^{3}Q_{3}E_{1}I_{1}-8Q_{1}E_{3}I_{3}a^{3}+8Q_{3}E_{1}I_{1}a^{3}+12LQ_{1}E_{3}I_{3}a^{2}-12LQ_{3}E_{1}I_{1}a^{2}}{24LE_{3}I_{3}}.$$

Finally, integration constants can be simplified into:

(43) $$c_{3}=-\frac{3L^{3}Q_{3}E_{1}I_{1}-8Q_{1}E_{3}I_{3}a^{3}+8Q_{3}E_{1}I_{1}a^{3}}{24LE_{1}I_{1}}$$

(44) $$c_{4}=\frac{9L^{3}Q_{3}E_{1}I_{1}-8Q_{1}E_{3}I_{3}a^{3}+8Q_{3}E_{1}I_{1}a^{3}}{24LE_{1}I_{1}}$$

(45) $$d_{3}=\frac{Q_{1}E_{3}I_{3}a^{3}-Q_{3}E_{1}I_{1}a^{3}}{3E_{1}I_{1}E_{3}I_{3}}$$

(46) $$d_{4}=-\frac{L^{3}Q_{3}E_{1}I_{1}-8Q_{1}E_{3}I_{3}a^{3}+8Q_{3}E_{1}I_{1}a^{3}}{24E_{1}I_{1}E_{3}I_{3}}.$$

### 3.3. Final Form of the Equation for AENF Deflection Curve by Euler–Bernoulli Beam Theory

Finally, four equations for AENF specimen deflections are derived:

(47) $$y_{\left[1\right]}^{SBT}=\frac{3L^{3}Q_{3}E_{1}I_{1}x-8Q_{1}E_{3}I_{3}a^{3}x+8Q_{3}E_{1}I_{1}a^{3}x+12LQ_{1}E_{3}I_{3}a^{2}x-12LQ_{3}E_{1}I_{1}a^{2}x-4LQ_{1}E_{3}I_{3}x^{3}}{24LE_{1}I_{1}E_{3}I_{3}}$$

(48) $$y_{\left[2\right]}^{SBT}=\frac{3L^{3}Q_{3}E_{2}I_{2}x-8Q_{2}E_{3}I_{3}a^{3}x+8Q_{3}E_{2}I_{2}a^{3}x+12LQ_{2}E_{3}I_{3}a^{2}x-12LQ_{3}E_{2}I_{2}a^{2}x-4LQ_{2}E_{3}I_{3}x^{3}}{24LE_{2}I_{2}E_{3}I_{3}}$$

(49) $$y_{\left[3\right]}^{SBT}=\frac{8LQ_{1}E_{3}I_{3}a^{3}-8LQ_{3}E_{1}I_{1}a^{3}-4LQ_{3}E_{1}I_{1}x^{3}+3L^{3}Q_{3}E_{1}I_{1}x-8Q_{1}E_{3}I_{3}a^{3}x+8Q_{3}E_{1}I_{1}a^{3}x}{24LE_{1}I_{1}E_{3}I_{3}}$$

(50) $$y_{\left[4\right]}^{SBT}=-\frac{\left(L-x\right)\left(L^{3}Q_{3}E_{1}I_{1}-8Q_{1}E_{3}I_{3}a^{3}+8Q_{3}E_{1}I_{1}a^{3}+4LQ_{3}E_{1}I_{1}x^{2}-8L^{2}Q_{3}E_{1}I_{1}x\right)}{24LE_{1}I_{1}E_{3}I_{3}}.$$

### 3.4. Crack Length as a Function of AENF Specimen Deflection

Equation (50) describes deflection of the beam in the 4th region as a function of abscissa x. Therefore, the crack length a can be determined as a function of material properties, specimen geometry, laminate configuration, and its deflection δ measured in central point $\delta =y_{\left[4\right]}^{SBT}\left(L/2\right)$, where:

(51) $$a=\sqrt[{3}]{\frac{LE_{1}I_{1}\left(Q_{3}L^{3}-9Q_{3}L^{2}x+12Q_{3}Lx^{2}-4Q_{3}x^{3}+24E_{3}I_{3}\delta \right)}{\left(8Q_{1}E_{3}I_{3}-8Q_{3}E_{1}I_{1}\right)\left(L-x\right)}}$$

which, for the half length of the specimen x=L/2 is:

(52) $$a=\sqrt[{3}]{\frac{24E_{1}I_{1}E_{3}I_{3}\delta -LQ_{3}E_{1}I_{1}}{4Q_{1}E_{3}I_{3}-4Q_{3}E_{1}I_{1}}}.$$

The specimen compliance is:

(53) $$C=\frac{\delta }{P}=\frac{\delta }{-2Q_{3}}=\frac{-\frac{\left(L-x\right)\left(L^{3}Q_{3}E_{1}I_{1}-8Q_{1}E_{3}I_{3}a^{3}+8Q_{3}E_{1}I_{1}a^{3}+4LQ_{3}E_{1}I_{1}x^{2}-8L^{2}Q_{3}E_{1}I_{1}x\right)}{24LE_{1}I_{1}E_{3}I_{3}}}{-2Q_{3}}$$

which is for the half specimen length, abscissa x=L/2:

(54) $$C^{\left(L/2\right)}=-\frac{L^{3}Q_{3}E_{1}I_{1}+4Q_{1}E_{3}I_{3}a^{3}-4Q_{3}E_{1}I_{1}a^{3}}{48Q_{3}E_{1}I_{1}E_{3}I_{3}}.$$

During the tests, the maximum compliance of the specimen occurs when a=L/2, while minimum when a=0:

(55) $$C_{max}^{\left(L/2\right)}=-\frac{L^{3}Q_{1}E_{3}I_{3}+L^{3}Q_{3}E_{1}I_{1}}{96Q_{3}E_{1}I_{1}E_{3}I_{3}}\;\mathrm{and}\;C_{min}^{\left(L/2\right)}=-\frac{L^{3}}{48E_{3}I_{3}}.$$

The negative sign indicates that the positive downward force causes negative beam deflection. The multiplication of compliance and downward force turn out into the well-known equation for beam deflection in a three-point bending test:

(56) $$\delta =\frac{PL^{3}}{48E_{3}I_{3}}.$$

## 4. Timoshenko Beam Theory Solution

### 4.1. Deflection of Two Timoshenko Cantilever Beams Fixed at One End

The deflections of two shear-deformable Timoshenko [13] cantilever beams can be written as:

(57) $$y_{\left[1\right]}^{TBT}=\frac{Q_{1}a^{3}}{3D_{1}}+\frac{Q_{1}a}{C_{1}}\;\mathrm{and}\;y_{\left[2\right]}^{TBT}=\frac{Q_{2}a^{3}}{3D_{2}}+\frac{Q_{2}a}{C_{2}}$$

where capital letters $C_{i}$ and $D_{i}$ respectively are the shear stiffness and bending stiffness of the *i*-th sublaminate. For homogeneous sublaminates, $C_{i}=\frac{5}{6}G_{i}A_{i}$ and $D_{i}=E_{i}I_{i}$, where $G_{i}$ and $E_{i}$ are the material shear modulus and Young modulus, $A_{i}$ and $I_{i}$ are the cross-section area and second moment of area, and 5/6 is the shear factor for a rectangular cross-section [21,22]. In general, such stiffnesses have to be calculated according to Classical Laminated Plate Theory [21], as specified in Appendix A.

The beam in the 1st and 2nd regions has the same deflection $y_{\left[1\right]}^{TBT}=y_{\left[2\right]}^{TBT}$, for the specified abscissa x=a:

(58) $$\frac{Q_{1}a^{3}}{3D_{1}}+\frac{Q_{1}a}{C_{1}}=\frac{Q_{2}a^{3}}{3D_{2}}+\frac{Q_{2}a}{C_{2}}.$$

Including Equation (2) and after a few simple calculations, the relation between the shear forces in the 1st, 2nd, and 3rd regions are derived:

(59) $$Q_{1}=Q_{3}\frac{\chi }{1+\chi }=\frac{\chi }{1+\chi }\frac{P}{2}\;\mathrm{and}\;Q_{2}=Q_{3}\frac{1}{1+\chi }=\frac{1}{1+\chi }\frac{P}{2}$$

where:

(60) $$\chi^{TBT}=\left(\frac{a^{2}}{3D_{2}}+\frac{1}{C_{2}}\right)/\left(\frac{a^{2}}{3D_{1}}+\frac{1}{C_{1}}\right).$$

Therefore, the known value of shearing force $Q_{3}$ acting on the 3rd region may be used to determine shearing forces $Q_{1}$ and $Q_{2}$ acting on the 1st and 2nd regions, and next, the equations for bending moments acting on these sublaminates:

(61) $$M_{1}\left(x\right)=Q_{1}x=\frac{\chi^{TBT}}{1+\chi^{TBT}}\frac{P}{2}x\;\mathrm{and}\;M_{2}\left(x\right)=Q_{2}x=\frac{1}{1+\chi^{TBT}}\frac{P}{2}x.$$

### 4.2. Deflection Curve of the AENF Specimen

#### 4.2.1. First Integrations of the Differential Equations for Beams Curvatures

According to the Timoshenko beam theory [13], the curvature of the *i*-th sublaminate, $\kappa_{\left[i\right]}$ is the derivative of the corresponding cross-section angle of rotation, $\phi_{\left[i\right]}$:

(62) $$\kappa_{\left[i\right]}\left(x\right)=\frac{d\phi_{\left[i\right]}}{dx}.$$

The bending moment is proportional to the curvature through the bending stiffness. Hence,

(63) $$M_{i}\left(x\right)=D_{i}\kappa_{\left[i\right]}\left(x\right)=D_{i}\frac{d\phi_{\left[i\right]}}{dx}.$$

Summarizing the above analyses, four differential equations are obtained for the four regions into which the AENF specimen is subdivided.

(64) $$\frac{d\phi_{\left[1\right]}}{dx}=\frac{M_{1}\left(x\right)}{D_{1}}=\frac{Q_{1}}{D_{1}}x$$

(65) $$\frac{d\phi_{\left[2\right]}}{dx}=\frac{M_{2}\left(x\right)}{D_{2}}=\frac{Q_{2}}{D_{2}}x$$

(66) $$\frac{d\phi_{\left[3\right]}}{dx}=\frac{M_{3}\left(x\right)}{D_{3}}=\frac{Q_{3}}{D_{3}}x$$

(67) $$\frac{d\phi_{\left[4\right]}}{dx}=\frac{M_{4}\left(x\right)}{D_{4}}=\frac{Q_{3}}{D_{3}}\left(L-x\right)$$

where the bending stiffness of a laminated beam in the 3rd and 4th regions is equal ($D_{4}=D_{3}$).

Now, the above-mentioned equations can be directly integrated to obtain the cross-section rotations:

(68) $$\phi_{\left[1\right]}\left(x\right)=\int^{\;}\frac{Q_{1}}{D_{1}}xdx=\frac{Q_{1}}{2D_{1}}x^{2}+c_{1}$$

(69) $$\phi_{\left[2\right]}\left(x\right)=\int \frac{Q_{2}}{D_{2}}xdx=\frac{Q_{2}}{2D_{2}}x^{2}+c_{2}$$

(70) $$\phi_{\left[3\right]}\left(x\right)=\int \frac{Q_{3}}{D_{3}}xdx=\frac{Q_{3}}{2D_{3}}x^{2}+c_{3}$$

(71) $$\phi_{\left[4\right]}\left(x\right)=\int \frac{Q_{3}}{D_{3}}\left(L-x\right)dx=\frac{Q_{3}}{D_{3}}x\left(L-\frac{1}{2}x\right)+c_{4}.$$

#### 4.2.2. First Boundary Condition: Cross-Section Rotations of the Beams at Singular Points 

The next step is to take into account the boundary conditions for the cross-section rotations at the joining points. Thus,

(72) $$\phi_{\left[1\right]}\left(a\right)=\phi_{\left[2\right]}\left(a\right)\;\mathrm{and}\;\phi_{\left[2\right]}\left(a\right)=\phi_{\left[3\right]}\left(a\right)\;\mathrm{and}\;\phi_{\left[3\right]}\left(\frac{L}{2}\right)=\phi_{\left[4\right]}\left(\frac{L}{2}\right)$$

which give:

(73) $$\frac{Q_{1}a^{2}}{2D_{1}}+c_{1}=\frac{Q_{2}a^{2}}{2D_{2}}+c_{2}$$

(74) $$\frac{Q_{2}a^{2}}{2D_{2}}+c_{2}=\frac{Q_{3}a^{2}}{2D_{3}}+c_{3}$$

(75) $$\frac{Q_{3}L^{2}}{8D_{3}}+c_{3}=\frac{3Q_{3}L^{2}}{8D_{3}}+c_{4}.$$

Hence,

(76) $$c_{2}=c_{1}+\frac{Q_{1}a^{2}}{2D_{1}}-\frac{Q_{2}a^{2}}{2D_{2}}$$

(77) $$c_{3}=c_{1}+\frac{Q_{1}a^{2}}{2D_{1}}-\frac{Q_{3}a^{2}}{2D_{3}}$$

(78) $$c_{4}=c_{1}+\frac{Q_{1}a^{2}}{2D_{1}}-\frac{Q_{3}a^{2}}{2D_{3}}-\frac{Q_{3}L^{2}}{4D_{3}}.$$

#### 4.2.3. Second Integration of the Beams Equations

For the *i*-th sublaminate modeled as a Timoshenko beam, the shear angle of cross-sections is

(79) $$\gamma_{i}\left(x\right)=\phi_{i}\left(x\right)+\frac{dy_{i}}{dx}=\frac{Q_{i}}{C_{i}}.$$

Hence, the deflection can be obtained by integrating the following equations

(80) $$\frac{dy_{i}^{TBT}}{dx}=\frac{Q_{i}}{C_{i}}-\phi_{i}\left(x\right).$$

By substituting the previous expressions, we get equations for four laminate regions:

(81) $$y_{\left[1\right]}^{TBT}\left(x\right)=\int \left[\frac{Q_{1}}{C_{1}}-\phi_{1}\left(x\right)\right]dx=\int \left[\frac{Q_{1}}{C_{1}}-\left(\frac{Q_{1}}{2D_{1}}x^{2}+c_{1}\right)\right]dx=-\frac{Q_{1}}{6D_{1}}x^{3}+\frac{Q_{1}}{C_{1}}x-c_{1}x+d_{1}$$

(82) $$y_{\left[2\right]}^{TBT}\left(x\right)=\int \left[\frac{Q_{2}}{C_{2}}-\phi_{2}\left(x\right)\right]dx=\int \left[\frac{Q_{2}}{C_{2}}-\left(\frac{Q_{2}}{2D_{2}}x^{2}+c_{2}\right)\right]dx=-\frac{Q_{2}}{6D_{2}}x^{3}+\frac{Q_{2}}{C_{2}}x-c_{2}x+d_{2}$$

(83) $$y_{\left[3\right]}^{TBT}\left(x\right)=\int \left[\frac{Q_{3}}{C_{3}}-\phi_{3}\left(x\right)\right]dx=\int \left[\frac{Q_{3}}{C_{3}}-\left(\frac{Q_{3}}{2D_{3}}x^{2}+c_{3}\right)\right]dx=-\frac{Q_{3}}{6D_{3}}x^{3}+\frac{Q_{3}}{C_{3}}x-c_{3}x+d_{3}$$

(84) $$y_{\left[4\right]}^{TBT}\left(x\right)=\int \left[\frac{Q_{4}}{C_{4}}-\phi_{4}\left(x\right)\right]dx=\int \left[-\frac{Q_{3}}{C_{3}}-\left(\frac{Q_{3}}{D_{3}}Lx-\frac{Q_{3}}{2D_{3}}x^{2}+c_{4}\right)\right]dx=\frac{Q_{3}}{6D_{3}}x^{3}-\frac{Q_{3}}{2D_{3}}Lx^{2}-\;\frac{Q_{3}}{C_{3}}x-c_{4}x+d_{4}.$$

#### 4.2.4. Second Boundary Conditions: Beam Deflections at Specimen Ends

The deflection at beam ends in the AENF test are zero, which enables specifying the following boundary conditions, for x=0:

(85) $$y_{\left[1\right]}^{TBT}\left(0\right)=-\frac{Q_{1}}{6D_{1}}0^{3}+\frac{Q_{1}}{C_{1}}0-c_{1}0+d_{1}=0\;\mathrm{and}\;y_{\left[2\right]}^{TBT}\left(0\right)=-\frac{Q_{2}}{6D_{2}}0^{3}+\frac{Q_{2}}{C_{2}}0-c_{2}0+d_{2}=0$$

which gives:

(86) $$d_{1}=0\;\mathrm{and}\;d_{2}=0$$

Similarly, for the second beam end, for x=L:

(87) $$y_{\left[4\right]}^{TBT}\left(L\right)=\frac{Q_{3}}{6D_{3}}L^{3}-\frac{Q_{3}}{2D_{3}}LL^{2}-\frac{Q_{3}}{C_{3}}L-c_{4}L+d_{4}=0$$

which gives:

(88) $$d_{4}=\frac{Q_{3}}{3D_{3}}L^{3}+\frac{Q_{3}}{C_{3}}L+c_{4}L.$$

#### 4.2.5. Third Boundary Conditions: Beam Deflections at Singular Points

Finally, the deflection of sublaminates at joining cross-sections must be equal:

(89) $$y_{\left[1\right]}^{TBT}\left(a\right)=y_{\left[2\right]}^{TBT}\left(a\right)\;\mathrm{and}\;y_{\left[2\right]}^{TBT}\left(a\right)=y_{\left[3\right]}^{TBT}\left(a\right)\;\mathrm{and}\;y_{\left[3\right]}^{TBT}\left(\frac{L}{2}\right)=y_{\left[4\right]}^{TBT}\left(\frac{L}{2}\right)$$

By substituting the previous expressions, we get

(90) $$-\frac{Q_{1}}{6D_{1}}a^{3}+\frac{Q_{1}}{C_{1}}a-c_{1}a+d_{1}=-\frac{Q_{2}}{6D_{2}}a^{3}+\frac{Q_{2}}{C_{2}}a-c_{2}a+d_{2}$$

(91) $$-\frac{Q_{2}}{6D_{2}}a^{3}+\frac{Q_{2}}{C_{2}}a-c_{2}a+d_{2}=-\frac{Q_{3}}{6D_{3}}a^{3}+\frac{Q_{3}}{C_{3}}a-c_{3}a+d_{3}$$

(92) $$-\frac{Q_{3}L^{3}}{48D_{3}}+\frac{Q_{3}L}{2C_{3}}-\frac{1}{2}c_{3}L+d_{3}=\frac{Q_{3}L^{3}}{48D_{3}}-\frac{Q_{3}L^{3}}{8D_{3}}-\frac{Q_{3}L}{2C_{3}}-\frac{1}{2}c_{4}L+d_{4}.$$

By recalling the previous expressions and solving, we obtain:

(93) $$\begin{array}{l} d_{2}=\frac{Q_{2}}{6D_{2}}a^{3}-\frac{Q_{1}}{6D_{1}}a^{3}+\frac{Q_{1}}{C_{1}}a-\frac{Q_{2}}{C_{2}}a-c_{1}a+c_{2}a\\ =\frac{Q_{2}}{6D_{2}}a^{3}-\frac{Q_{1}}{6D_{1}}a^{3}+\frac{Q_{1}}{C_{1}}a-\frac{Q_{2}}{C_{2}}a-c_{1}a+(c_{1}+\frac{Q_{1}a^{2}}{2D_{1}}-\frac{Q_{2}a^{2}}{2D_{2}})a\\ =\frac{P}{2}\frac{a}{1+\chi }\left[\chi \left(\frac{a^{2}}{3D_{1}}+\frac{1}{C_{1}}\right)-\left(\frac{a^{2}}{3D_{2}}+\frac{1}{C_{2}}\right)\right]\\ =\frac{P}{2}\frac{a}{1+\chi }\left[\frac{\frac{a^{2}}{3D_{2}}+\frac{1}{C_{2}}}{\frac{a^{2}}{3D_{1}}+\frac{1}{C_{1}}}\left(\frac{a^{2}}{3D_{1}}+\frac{1}{C_{1}}\right)-\left(\frac{a^{2}}{3D_{2}}+\frac{1}{C_{2}}\right)\right]=0 \end{array}$$

which we already had obtained; actually, the above equation is redundant having posed $y_{\left[1\right]}^{TBT}=y_{\left[2\right]}^{TBT}$.

(94) $$\begin{array}{l} d_{3}=\frac{Q_{3}}{6D_{3}}a^{3}-\frac{Q_{2}}{6D_{2}}a^{3}+\frac{Q_{2}}{C_{2}}a-\frac{Q_{3}}{C_{3}}a-c_{2}a+c_{3}a\\ =\frac{Q_{3}}{6D_{3}}a^{3}-\frac{Q_{2}}{6D_{2}}a^{3}+\frac{Q_{2}}{C_{2}}a-\frac{Q_{3}}{C_{3}}a-\left(c_{1}+\frac{Q_{1}a^{2}}{2D_{1}}-\frac{Q_{2}a^{2}}{2D_{2}}\right)a\\ +\left(c_{1}+\frac{Q_{1}a^{2}}{2D_{1}}-\frac{Q_{3}a^{2}}{2D_{3}}\right)a=Q_{2}a\left(\frac{a^{2}}{3D_{2}}+\frac{1}{C_{2}}\right)-Q_{3}a\left(\frac{a^{2}}{3D_{3}}+\frac{1}{C_{3}}\right)\\ =\frac{P}{2}a\left[\frac{1}{1+\chi }\left(\frac{a^{2}}{3D_{2}}+\frac{1}{C_{2}}\right)-\left(\frac{a^{2}}{3D_{3}}+\frac{1}{C_{3}}\right)\right]\\ =\frac{P}{2}a\left(\frac{1}{\frac{1}{\frac{a^{2}}{3D_{1}}+\frac{1}{C_{1}}}+\frac{1}{\frac{a^{2}}{3D_{2}}+\frac{1}{C_{2}}}}-\frac{a^{2}}{3D_{3}}-\frac{1}{C_{3}}\right) \end{array}$$

and

(95) $$d_{4}=-\frac{Q_{3}L^{3}}{48D_{3}}+\frac{Q_{3}L}{2C_{3}}-\frac{1}{2}c_{3}L+d_{3}-\frac{Q_{3}L^{3}}{48D_{3}}+\frac{Q_{3}L^{3}}{8D_{3}}+\frac{Q_{3}L}{2C_{3}}+\frac{1}{2}c_{4}L=\frac{Q_{3}L^{3}}{12D_{3}}+\frac{Q_{3}L}{C_{3}}-\frac{1}{2}\left(c_{1}+\frac{Q_{1}a^{2}}{2D_{1}}-\frac{Q_{3}a^{2}}{2D_{3}}\right)L\;+\frac{1}{2}\left(c_{1}+\frac{Q_{1}a^{2}}{2D_{1}}-\frac{Q_{3}a^{2}}{2D_{3}}-\frac{Q_{3}L^{2}}{4D_{3}}\right)L+d_{3}=-\frac{Q_{3}L^{3}}{24D_{3}}+\frac{Q_{3}L}{C_{3}}+d_{3}=\frac{P}{2}\left(\frac{a}{\frac{1}{\frac{a^{2}}{3D_{1}}+\frac{1}{C_{1}}}+\frac{1}{\frac{a^{2}}{3D_{2}}+\frac{1}{C_{2}}}}-\frac{L^{3}+8a^{3}}{24D_{3}}+\frac{L-a}{C_{3}}\right).$$

By recalling the previous expressions of $c_{4}$ and $d_{4}$,

(96) $$c_{1}=\frac{Q_{3}a^{2}}{2D_{3}}-\frac{Q_{3}L^{2}}{12D_{3}}-\frac{Q_{3}}{C_{3}}-\frac{Q_{1}a^{2}}{2D_{1}}+\frac{d_{4}}{L}\;=\frac{P}{2L}\left[\frac{12La^{2}-3L^{3}-8a^{3}}{24D_{3}}-\frac{\chi }{1+\chi }\frac{La^{2}}{2D_{1}}+\frac{1}{1+\chi }a\left(\frac{a^{2}}{3D_{2}}+\frac{1}{C_{2}}\right)-\frac{a}{C_{3}}\right]$$

(97) $$c_{2}=\frac{P}{2L}[\frac{12La^{2}-3L^{3}-8a^{3}}{24D_{3}}-\frac{\chi }{1+\chi }\frac{La^{2}}{2D_{1}}+\frac{1}{1+\chi }a\left(\frac{a^{2}}{3D_{2}}+\frac{1}{C_{2}}\right)-\frac{a}{C_{3}}]+\frac{Q_{1}a^{2}}{2D_{1}}-\frac{Q_{2}a^{2}}{2D_{2}}\;=\frac{P}{2L}[\frac{12La^{2}-3L^{3}-8a^{3}}{24D_{3}}+\frac{a}{1+\chi }\left(\frac{a^{2}}{3D_{2}}-\frac{La}{2D_{2}}+\frac{1}{C_{2}}\right)-\frac{a}{C_{3}}]$$

(98) $$c_{3}=\frac{P}{2L}[\frac{12La^{2}-3L^{3}-8a^{3}}{24D_{3}}-\frac{\chi }{1+\chi }\frac{La^{2}}{2D_{1}}+\frac{1}{1+\chi }a\left(\frac{a^{2}}{3D_{2}}+\frac{1}{C_{2}}\right)-\frac{a}{C_{3}}]+\frac{Q_{1}a^{2}}{2D_{1}}-\frac{Q_{3}a^{2}}{2D_{3}}\;=\frac{P}{2L}\left[-\frac{3L^{3}+8a^{3}}{24D_{3}}+\frac{a}{1+\chi }\left(\frac{a^{2}}{3D_{2}}+\frac{1}{C_{2}}\right)-\frac{a}{C_{3}}\right]$$

(99) $$c_{4}=\frac{P}{2L}[\frac{12La^{2}-3L^{3}-8a^{3}}{24D_{3}}-\frac{\chi }{1+\chi }\frac{La^{2}}{2D_{1}}+\frac{1}{1+\chi }a\left(\frac{a^{2}}{3D_{2}}+\frac{1}{C_{2}}\right)-\frac{a}{C_{3}}]+\frac{Q_{1}a^{2}}{2D_{1}}-\frac{Q_{3}a^{2}}{2D_{3}}-\frac{Q_{3}L^{2}}{4D_{3}}=\;\frac{P}{2L}[-\frac{9L^{3}+8a^{3}}{24D_{3}}+\frac{a}{1+\chi }\left(\frac{a^{2}}{3D_{2}}+\frac{1}{C_{2}}\right)-\frac{a}{C_{3}}].$$

#### 4.2.6. Final Form of the Equation for AENF Deflection Curve by Timoshenko Beam Theory

Including integration constants into Equations (81)–(84), the final equation for the AENF specimen deflection profile has been obtained. The equations are presented in short 1-line form (to be directly copied into the calculation software) below:

(100) $$\begin{array}{ll} y_{\left[1\right]}^{TBT}\left(x\right)= & \\ (P\cdot x\cdot (a/C_{3}+ & (3\cdot L^{3}-12\cdot L\cdot a^{2}+8\cdot a^{3})/\left(24\cdot D_{3}\right)-(a\cdot \left(1/C_{2}+a^{2}/\left(3\cdot D_{2}\right))\right)/\left(\chi^{TBT}+1\right)\\ & +\left(L\cdot \chi^{TBT}\cdot a^{2}\right)/\left(2\cdot D_{1}\cdot \left(\chi^{TBT}+1\right)\right)))/\left(2\cdot L\right)+\left(P\cdot \chi^{TBT}\cdot x\right)/(2\cdot C_{1}\cdot \left(\chi^{TBT}+1)\right)\\ & -\left(P\cdot \chi^{TBT}\cdot x^{3}\right)/\left(12\cdot D_{1}\cdot \left(\chi^{TBT}+1\right)\right) \end{array}$$

(101) $$\begin{array}{ll} y_{\left[2\right]}^{TBT}\left(x\right)= & \\ (P\cdot x\cdot (a/C_{3}+ & \left(3\cdot L^{3}-12\cdot L\cdot a^{2}+8\cdot a^{3}\right)/\left(23\cdot D_{3}\right)-(a\cdot (1/C_{2}+a^{2}/\left(3\cdot D_{2}\right)-\left(L\cdot a\right)/(2\\ & \cdot D_{2})))/\left(\chi^{TBT}+1\right)))/\left(2\cdot L\right)-\left(P\cdot x^{3}\right)/\left(12\cdot D_{2}\cdot \left(\chi^{TBT}+1\right)\right)+\left(P\cdot x\right)/(2\\ & \cdot C_{2}\cdot \left(\chi^{TBT}+1\right)) \end{array}$$

(102) $$\begin{array}{ll} y_{\left[3\right]}^{TBT}\left(x\right)= & \\ \left(P\cdot x)/(2\cdot C_{3}\right) & -(P\cdot x^{3})/(12\cdot D_{3})-(P\cdot a\cdot (1/C_{3}-\left(1/C_{2}+a^{2}/(3\cdot D_{2}))/(\chi^{TBT}+1\right)\\ & +a^{2}/\left(3\cdot D_{3}\right)))/2+(P\cdot x\cdot (a/C_{3}+\left(3\cdot L^{3}+8\cdot a^{3})/(24\cdot D_{3}\right)-(a\cdot (1/C_{2}\\ & +a^{2}/\left(3\cdot D_{2}\right)))/\left(\chi^{TBT}+1\right)))/\left(2\cdot L\right) \end{array}$$

(103) $$\begin{array}{l} y_{\left[4\right]}^{TBT}\left(x\right)=\\ (P\cdot (\left(L-a\right)/C_{3}-\left(L^{3}+8\cdot a^{3}\right)/\left(24\cdot D_{3}\right)+a/\left(1/(1/(C_{1}+a^{2}/(3\cdot D_{1})\right)+1/(1/(C_{2}+a^{2}/(3\cdot \\ D_{2})))))/2+\left(P\cdot x^{3}\right)/\left(12\cdot D_{3}\right)-(P\cdot x)/(2\cdot C_{3})-(L\cdot P\cdot x^{2})/\left(4\cdot D_{3}\right)+(P\cdot x\cdot (a/C_{3}+(9\cdot \\ L^{3}+8\cdot a^{3})/(24\cdot D_{3})-\left(a\cdot \left(1/C_{2}+a^{2}/\left(3\cdot D_{2}\right)\right)\right)/\left(\chi^{TBT}+1\right)))/\left(2\cdot L\right). \end{array}$$

**Figure 5:**
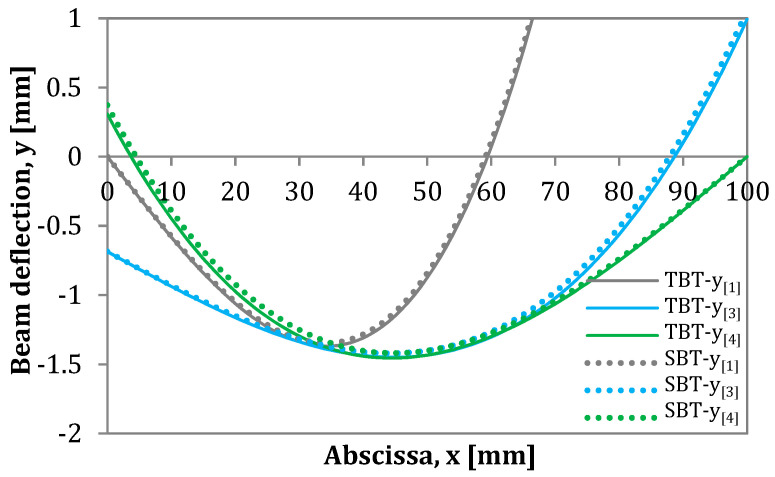
Deflection curves for the four regions of the loaded AENF specimen.

Below in Figure 5, an example of the AENF specimen deflection curve is presented derived from the above four equations.

The deflection under the load application point turns out to be

(104) $$\delta =y_{\left[4\right]}^{TBT}\left(\frac{L}{2}\right)=\frac{Q_{3}}{48D_{3}}L^{3}-\frac{Q_{3}}{8D_{3}}L^{3}-\frac{Q_{3}L}{2C_{3}}-\frac{c_{4}L}{2}+d_{4}=\frac{PL^{3}}{48D_{3}}-\frac{Pa^{3}}{12D_{3}}+\frac{p}{4}\frac{a}{1+\chi }\left(\frac{a^{2}}{3D_{2}}+\frac{1}{C_{2}}\right)+\frac{p}{4}\frac{L-a}{4C_{3}}=\;\frac{P}{4}\left[\frac{L^{3}-4a^{3}}{12D_{3}}+\frac{a}{1+\chi }\left(\frac{a^{2}}{3D_{2}}+\frac{1}{C_{2}}\right)+\frac{L-a}{C_{3}}\right].$$

The specimen compliance is:

(105) $$C=\frac{\delta }{P}=\frac{L^{3}-4a^{3}}{48D_{3}}+\frac{1}{4}\frac{a}{1+\chi }\left(\frac{a^{2}}{3D_{2}}+\frac{1}{C_{2}}\right)+\frac{L-a}{4C_{3}}.$$

For a symmetric and homogeneous specimen,

(106) $$\chi =1,\;C_{2}=\frac{5}{6}GBh,\;D_{2}=\frac{1}{12}EBh^{3},\;C_{3}=2C_{2},\;\mathrm{and}\;D_{3}=8D_{2},$$

where *h* is the specimen half thickness, *G* is a transversal shearing modulus, and *B* is the specimen width. Thus, the compliance becomes:

(107) $$C=\frac{\delta }{P}=\frac{L^{3}}{384D_{2}}+\frac{a^{3}}{32D_{2}}+\frac{L}{8C_{2}}=\frac{L^{3}+12a^{3}}{32EBh^{3}}+\frac{3L}{20GBh}=\frac{2l^{3}+3a^{3}}{8EBh^{3}}+\frac{3l}{10GBh}$$

where l=L/2 is the specimen half length. This expression is consistent with the well-known expression in the literature [14]. 

For *a* = 0, we obtain the well-known expression of a beam deflection in a three-point bending test:

(108) $$\delta =\frac{PL^{3}}{48D_{3}}+\frac{PL}{4C_{3}}.$$

If the specimen compliance $C_{exp}$ is measured by an experiment, we know $C_{exp}=\delta_{exp}/P_{exp}$. Thus, the crack length can be estimated by solving the following cubic equation:

(109) $$\frac{1}{3}\left(\frac{1}{1+\chi }\frac{1}{D_{2}}-\frac{1}{D_{3}}\right)a^{3}+\left(\frac{1}{1+\chi }\frac{1}{C_{2}}-\frac{1}{C_{3}}\right)a+12\frac{L^{3}}{D_{3}}+\frac{L}{C_{3}}-4C_{exp}=0.$$

Values can be obtained numerically [23] or by using the analytical formulas [24]. The presented methodology can be used to estimate the actual crack length as a function of specimen compliance. Such analytical equations are needed in particular for the determination of a posteriori crack length during fatigue mode II tests, since the macroscopic observations are difficult and burdened with a significant error due to the formation micro cracks near the major crack tip [25,26,27,28]. 

## 5. Experimental and Numerical Validation

### 5.1. Methodology

A series of experimental tests on AENF specimens were performed. During the displacement controlled tests, deflection of the central point was recorded as a function of downward force *P_d_* acting on the central point of the specimen. Subsequently, the specimen compliance was determined for different layup configurations. Experimental tests were performed on a Shimadzu ASX Plus (Kyoto, Japan) with a load capacity of 20 kN. The specimen’s geometry has been the same in both experimental and numerical tests. All of the manufactured specimens have been cut from the larger delaminated plate, where the final width *B* of the specimens has been measured by caliper and was equal to *B* = 23.5 mm in all of the cases. A fixture span *L* = 100 mm was used during the tests with a displacement rate of 0.5 mm/min.

**Figure 6:**
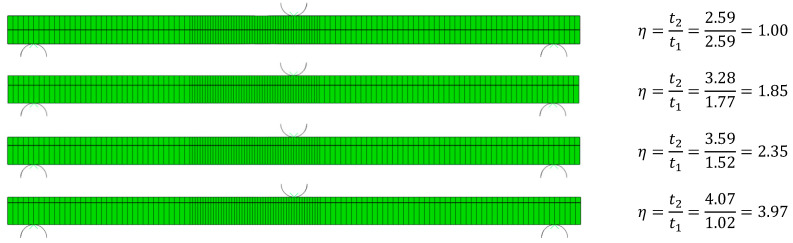
Four cases of numerically simulated laminates for comparison with analytical solution.

The analytical solutions derived in previous sections SBT and TBT were used to determine the deflection curves of centrally loaded AENF specimens, which were then compared with a numerical model in ABAQUS/Standard software. Four cases of glass fibre-reinforced laminates have been modeled. In numerical simulations, a standard and directly available in ABAQUS, model of lamina in plane-stress was used, based on FSDT. A continuum of shell elements was used with only three integration points through the thickness. The interface between the top and bottom sublaminate was modeled by cohesive elements with a thickness of 0.01 mm. The properties of cohesive elements for the GFRP interface in direction 0°//0° can be found in reference [8]. A rectangular mesh with a size of 1 mm was applied into the model, while in the crack tip area, the mesh size was refined into 0.5 mm, as shown in Figure 6. Four laminates have been considered—one symmetrical and three asymmetrical with different asymmetry ratios $\eta =t_{2}/t_{1}$, where $t_{1}$ and $t_{2}$ are the thickness of sublaminate 1 and sublaminate 2, respectively.

**Figure 7:**
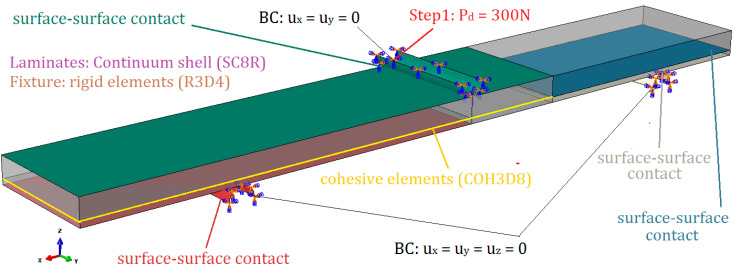
Boundary conditions and types of finite element applied in the numerical model.

The numerical simulations have been performed on GFRP laminates with unidirectional orientation. The following material properties have been employed: longitudinal Young modulus *E*_[i]_ = *E_x_* = 47,057 MPa; perpendicular Young modulus *E_y_* = 14,920 MPa; transversal shear modulus in plane xy, *G_xy_* = 5233 MPa; transversal shear modulus in plane xz, *G*_[i]_ = *G_xz_* = 5233 MPa; transversal shear modulus in plane yz, *G_yz_* = 4000 MPa; Poisson ratio in plane xy, *ν_xy_* = 0.27. In the simulations, the symmetric and asymmetric specimens with delamination length a=30 mm have been loaded by downward force $P_{d}=300\;N$. The boundary conditions as well as types of finite elements applied in numerical model are presented in Figure 7.

### 5.2. Deflection Profiles of the ENF and AENF Specimen

**Figure 8:**
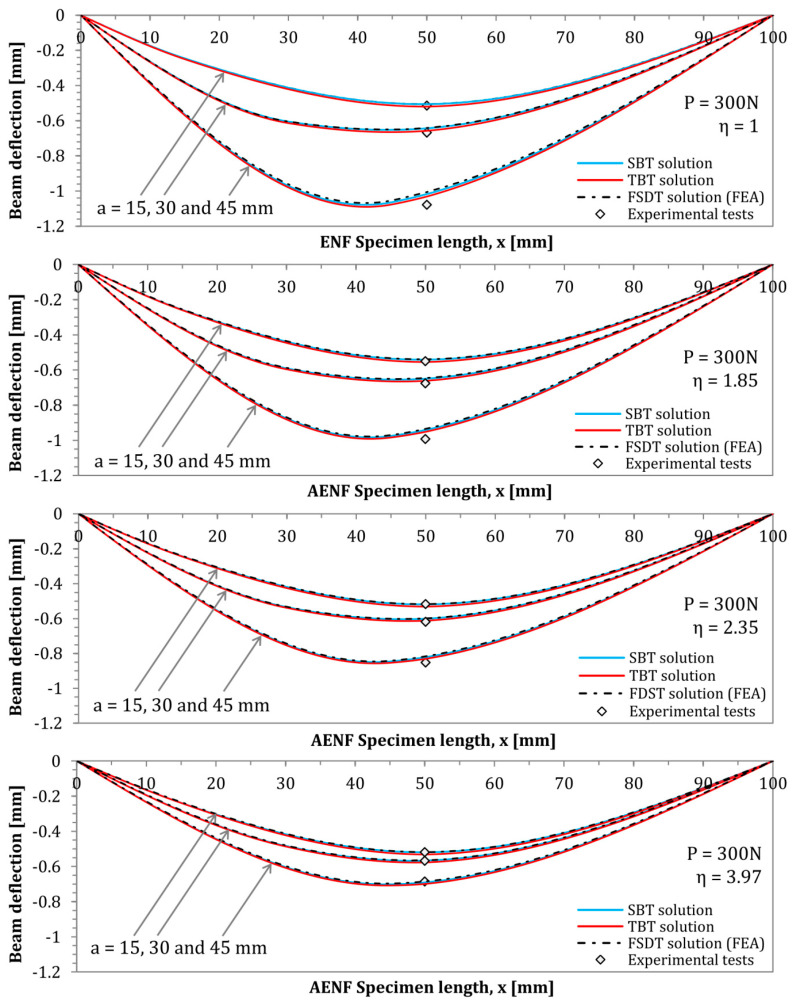
AENF test specimen deflection profiles derived by three different methods and test results.

The results obtained by using the SBT model, the TBT, the numerical finite element model (FSDT), as well as the experimentally determined value of the beam deflection (at the central point, for x=50 mm) are presented in Figure 8. 

The experimentally determined during CC tests deflection *δ_EXP_* of analyzed laminates as well as numerical *δ_FEA_* and analytical modeling *δ_TBT_* and *δ_SBT_* results for different crack lengths are presented in Table 1.

**Figure 9:**
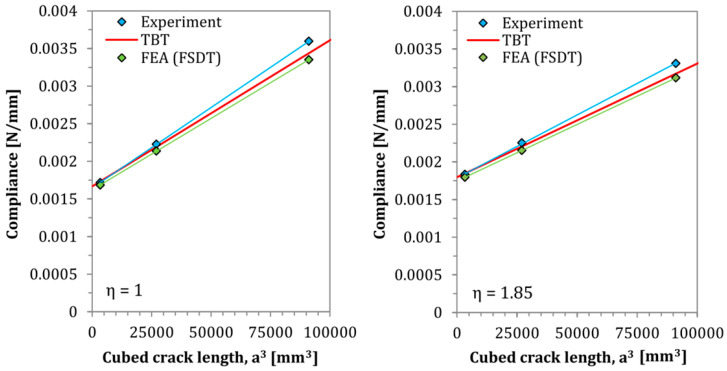
Predicted and tested compliances of homogeneous laminates η = 1 and η = 1.85.

**Figure 10:**
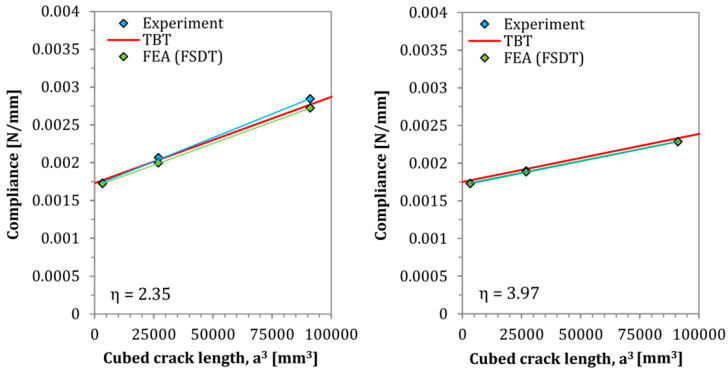
Predicted and tested compliances of homogeneous laminates η = 2.35 and η = 3.97.

The results of the theoretically performed compliance–calibration tests based on the derived TBT model are presented in Figure 9 and Figure 10.

As can be observed in Figure 8, Figure 9 and Figure 10, as well as in Table 1, a very good matching of results is obtained between the analytical and numerical models, and the experimental tests. In all cases, deflection profiles obtained by the SBT model exhibited slightly lower deflections than the modeled by the TBT. These differences are clearly caused by the inclusion of shear deformability. The FSDT solution obtained by Finite Element Analysis yielded deflection curves with a shape almost perfectly matched the analytical solutions. The reason for some very small mismatch between their deflections could be the slight interpenetration of finite elements of rigid support and elastic composite elements in numerical model. In addition, regarding the theoretically performed compliance–calibration tests, the derived TBT model for Asymmetrical End Notch Flexure was marked by very good accuracy, while an even better fit with experimental tests has been obtained than by finite element numerical analyses, which are burdened with contact imperfections between nodes of rigid fixture support and elastic deformed AENF laminated specimen. 

## 6. Influence of Cross-Section Rotation on the Internal Forces in AENF

### 6.1. Parametric Study-Simple vs. Timoshenko Beam Theory

**Figure 11:**
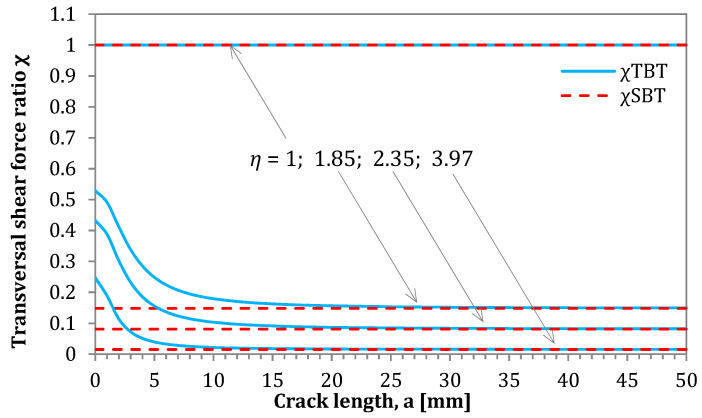
Transversal shearing forces ratio χ in the delaminated part of the AENF specimen.

In this section, the influence of applied theories on the magnitude of loads acting on the AENF specimen as a function of crack length was analyzed. The SBT and TBT models, as well as the numerical model FSDT were used for the determination of shearing forces *Q*_1_ and *Q*_2_ acting in the 1st and 2nd region of AENF specimen. The comparison is shown in Figure 11. 

**Figure 12:**
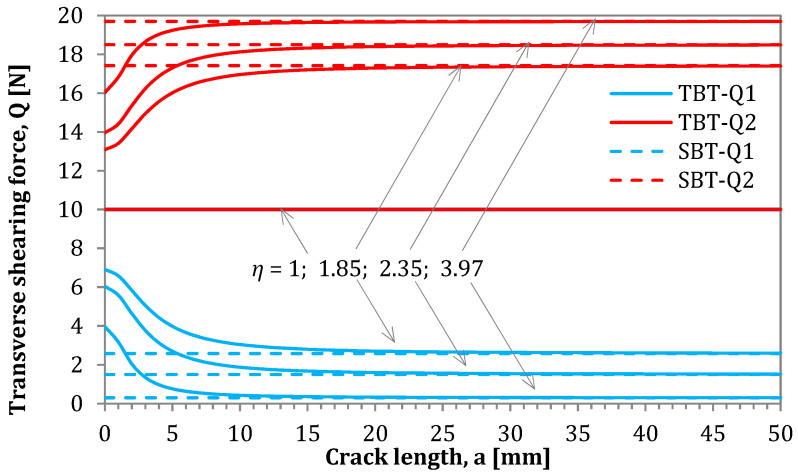
Magnitudes of transversal shearing forces in the delaminated part of the AENF specimen.

In the presented solution for the determination of transversal shearing forces based on SBT, the parameter $\chi_{SBT}$ may be determined according to Equation (12). As can be seen in the above-mentioned equation and Figure 9, such parameter is crack length independent, and its magnitude is controlled only by the stiffness ratio between the 1st and 2nd region of the AENF specimen. A different situation occurs for parameter $\chi_{TBT}$ derived from TBT and described by Equation (60). The influence of the shearing force ratio parameters $\chi_{SBT}$ and $\chi_{TBT}$ on the magnitudes of transversal shearing forces *Q*_1_ and *Q*_2_ acting on the 1st and 2nd regions of the AENF specimen is shown in Figure 12.

It may be observed in Figure 11 that the χ parameter is crack-length dependent, when cross-section rotation is kinematically allowed. For short cracks, the transition of transversal shearing force Q from the 3rd region of the AENF (undelaminated part of the beam) into two delaminated 1st and 2nd beams are rather smooth and more uniformly distributed than when using in SBT solution. On the contrary, the application of SBT yields sudden shearing force jumps in the vicinity of the crack tip. However, it can be also clearly seen from Figure 12 that the transversal shearing forces calculated by TBT tends to the values determined by SBT with the increase of crack length a. Thus, for relatively long cracks (a>10 mm), the values of shearing forces calculated by SBT and TBT yield similar results. The above observation indicates that blocking (SBT) or enabling (TBT) rotations of the laminate cross-section are crucial for the shearing forces magnitude near the crack tip, especially for the short cracks (a<10 mm). 

### 6.2. Three-Dimensional Finite Element Analysis Modeling of AENF

In this section, the numerical simulation results of the three-point bending test performed on AENF specimens are presented. In the below figures, the internal forces in the sublaminate at the top of delamination have been presented on the left, while the sublaminate at the bottom of delamination is shown on the right.

**Figure 13:**
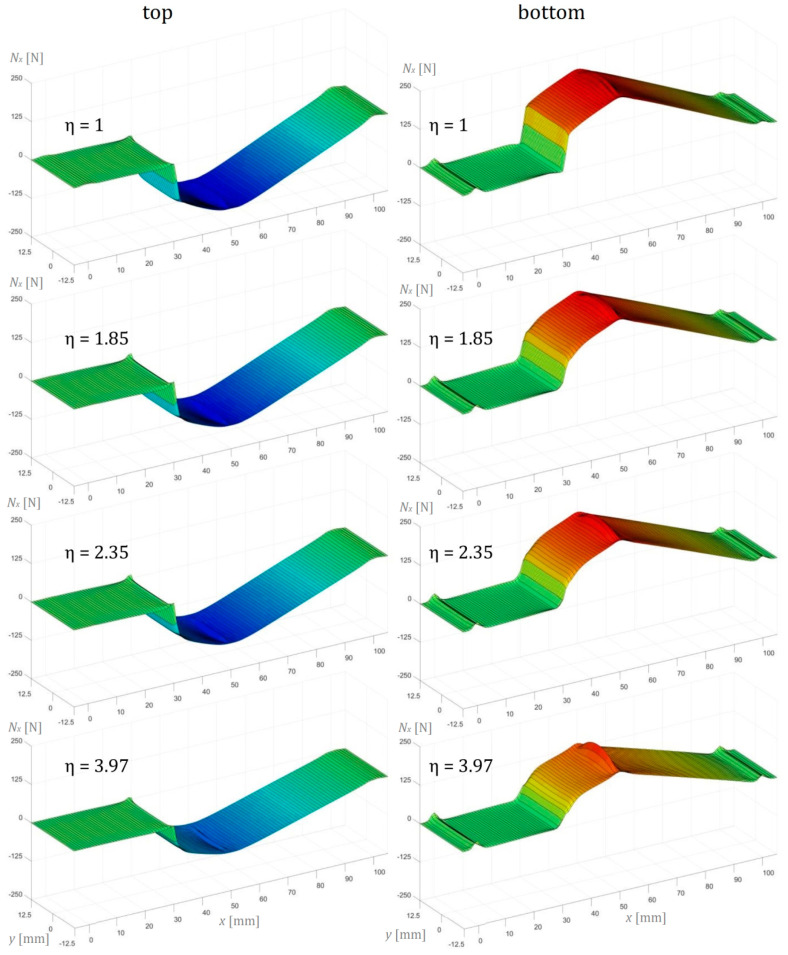
Normal forces per unit width in the longitudinal direction *N_x_* of the AENF.

As can be observed in Figure 13, the normal forces in the longitudinal direction *N_x_* are present only in the non-cracked region of the AENF specimen. In addition, the effect of fixture indenter is visible in the central point of the span, causing additional tension in the bottom sublaminate. A similar but two-dimensional analytically derived evolution of internal forces in the top and lower sublaminates was presented by Jumel et al. [29] for the symmetric ENF made of isotropic homogenous adherents. Jumel et al. [29] and Budzik et al. [30] observed that the internal forces are changing from a pure bending moment in the delaminated part of the specimen into complex tension, compression, and bending in the bonded region.

**Figure 14:**
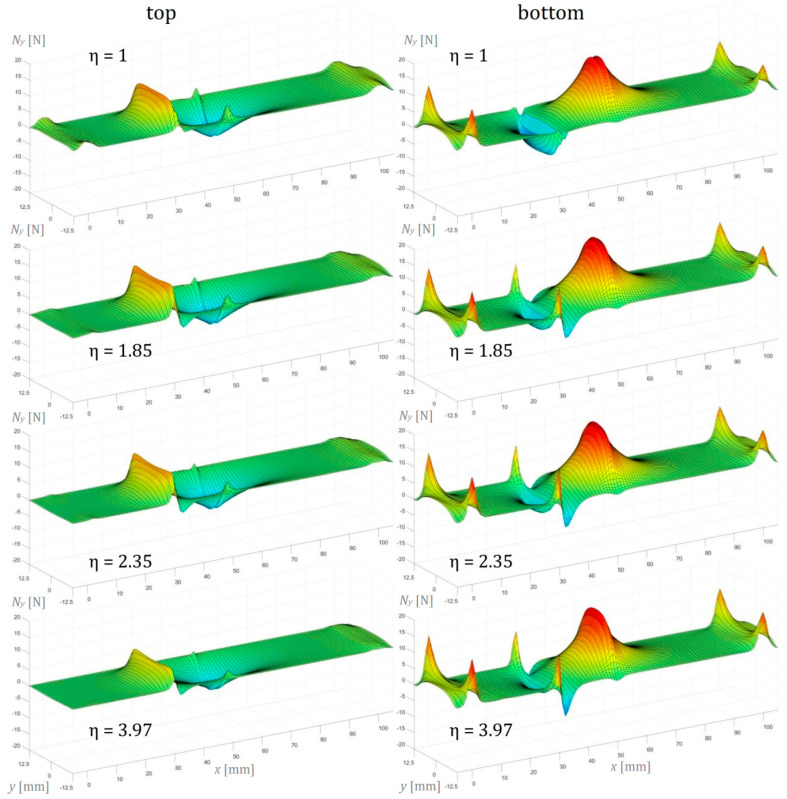
Normal forces per unit width in the transversal direction *N_y_* of the AENF.

Conversely to the longitudinal normal forces *N_x_*, the normal forces in the perpendicular direction *N_y_* are rather small and have not exceeded 20 N (Figure 14). However, their distribution is more non-uniform, exhibiting significant fluctuations especially on the edge at the vicinity of the crack tip (for x=30 mm), as well as in the regions of the specimen in contact with the fixture support and central indenter. It should be noted that in the top adherent, the globally compression forces induced by the central indenter evolve into the tension in the delaminated part, which is close to the crack tip. Such effect seems to be rather independent of the unidirectional laminate configuration, exhibiting similar behavior for symmetrical (η=1) and asymmetrical laminates (η>1).

**Figure 15:**
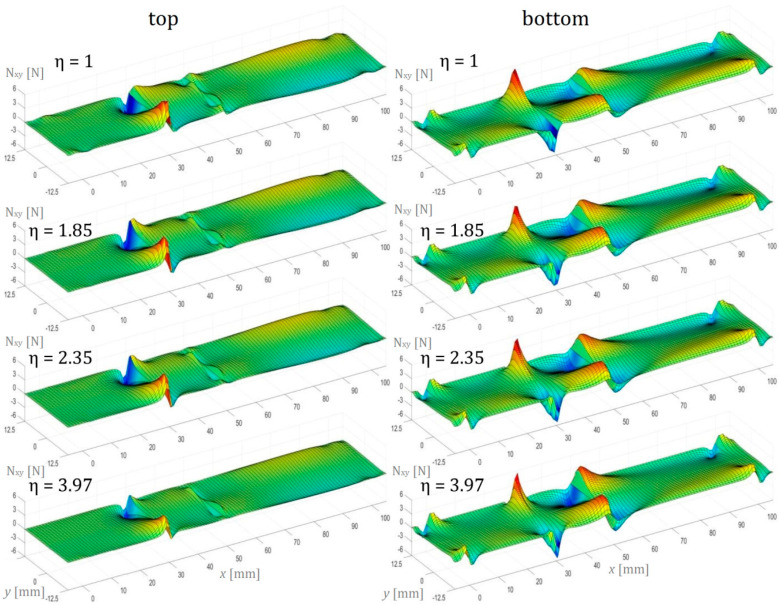
In-plane shear forces N_xy_ per unit width in the AENF.

In Figure 15, the magnitude of in-plane shear membrane forces N_xy_ per unit width in the global xy plane of the top and bottom beam was shown. As can N_xy_ be observed, the shear forces in the loaded AENF specimen are asymmetric with respect to the two directions. A similar asymmetrical distribution of in-plane shearing forces over the interface of the undelaminated region was observed by Szekrenyes [31] in a carbon/epoxy multidirectional delaminated plate. As concluded by Szekrenyes [31], the fluctuations related to the interfacial shear stresses resulted in a significant mode III contribution in the energy release rate distributions at the crack tip. However, as may be seen in Figure 14, the anti-planar shear (mode III) contribution concentrated at the AENF undelaminated edge over the crack tip is present also in the unidirectional laminates, even in the case of symmetrical configuration.

**Figure 16:**
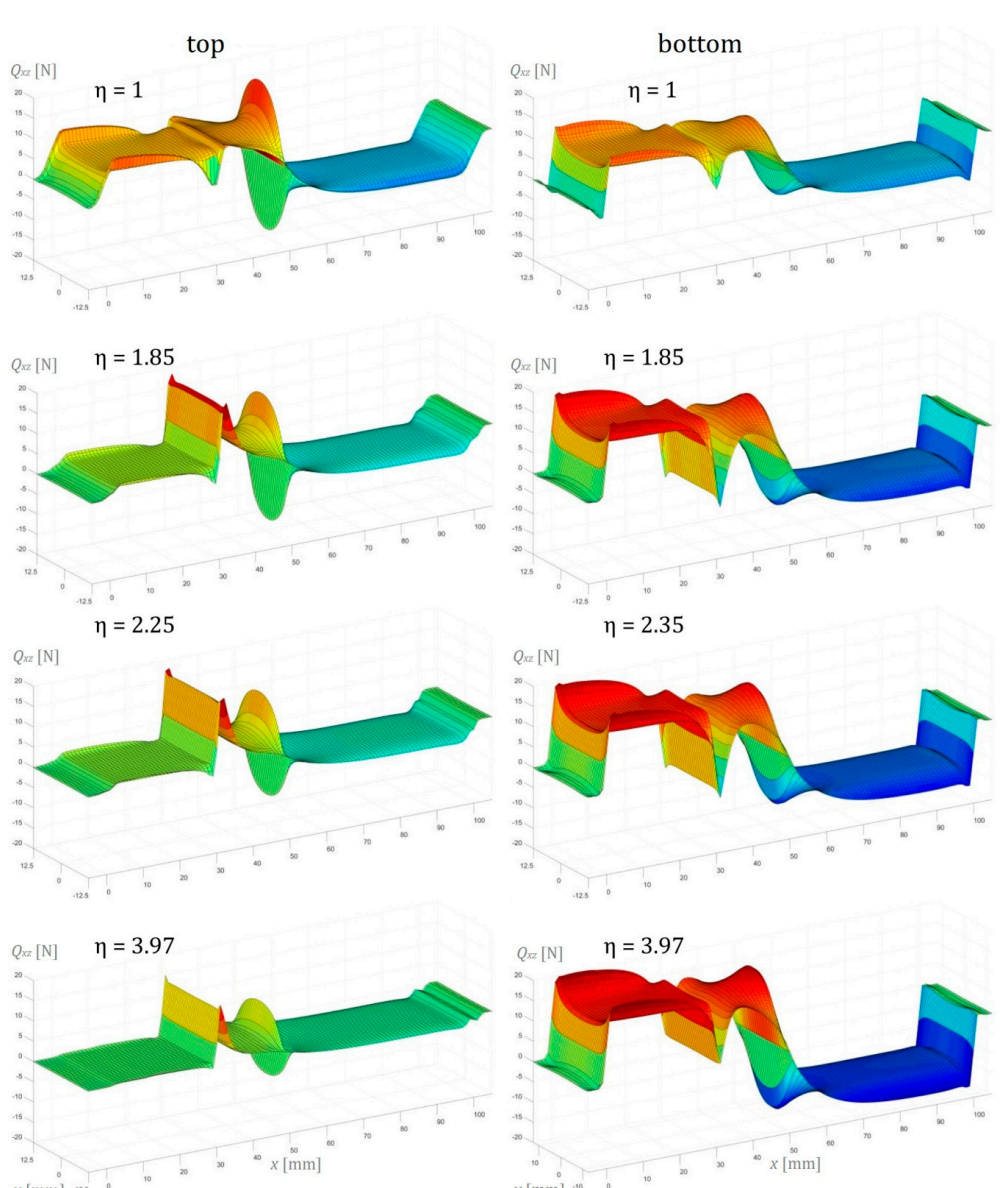
Transverse shear force per unit width *Q_xz_* in the longitudinal direction of the AENF.

**Figure 17:**
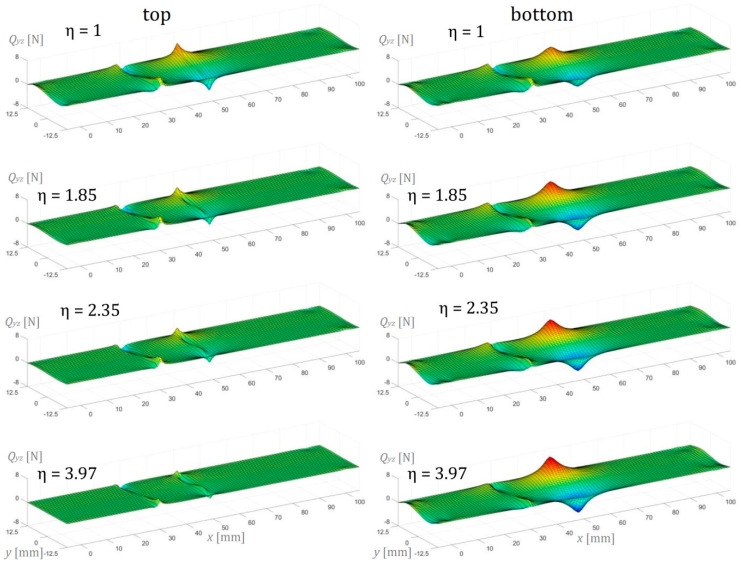
Transverse shear force per unit width *Q_yz_* in the perpendicular direction of the AENF.

The transversal shearing forces in the top and bottom sublaminate determined by Finite Element Analysis are presented in Figure 16 in the transversal-longitudinal plane (*Q_xz_*) and in Figure 17 for the transversal-perpendicular plane (*Q_yz_*). The effect of indenter is clearly visible in the top sublaminate and according to the SBT and TBT [13], it vanishes directly below the central loading point (for x=50 mm) in three-point bending configuration. According to Jumel et al. [29], the large shear forces gradient in the front of the crack may be associated with the conversion of the internal force from the pure bending moment in the delaminated regions of the specimen (sublaminate 1 and 2) into combined bending with tensile and compression stresses in the undelaminated part of the beam (vicinity of the crack tip in sublaminate 3). As performed in current research, a 3D numerical analysis revealed also the edge effect in the transversal shearing force distribution (Figure 16), suggesting that the increase of the shearing force at the delaminated edge may be caused by the anticlastic curvature of the deformed AENF beam. In the case of symmetrical configuration (ENF), the distribution of transversal shearing forces at the crack tip area was rather similar in sublaminate on the top and at the bottom of the delamination plane. However, in the case of asymmetric configurations (AENF), the transversal shear forces suddenly increase and decrease just ahead of the crack tip in the top and bottom laminate respectively, which may affect also the mode mixity at the crack tip in the AENF unidirectional specimens [29,30].

**Figure 18:**
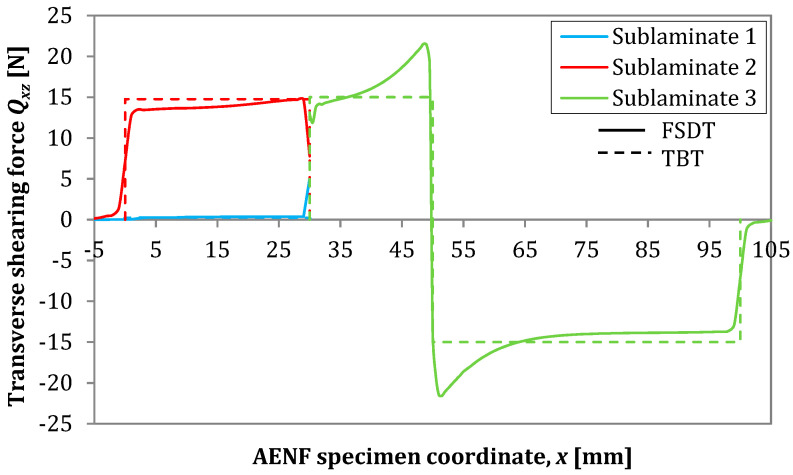
Transversal shear force *Q_xz_* distribution by analytical Timoshenko beam theory (TBT) and numerical first-order shear deformation theory (FSDT) model.

The transversal shearing forces in the transversal–perpendicular plane (*Q_yz_*) were about four times lower than those for the longitudinal direction. However, also in this case, some inhomogeneous distribution may be observed especially near the crack tip area (Figure 17). A comparison of the transversal shearing forces distribution over the specimen span determined by the TBT analytical model as well as finite element analysis is presented in Figure 18.

As can be observed in Figure 18, a good agreement has been obtained between the results of analytical modeling (TBT) and FSDT. However, the numerical analyses ensure more refined force field in the AENF specimen, especially near the crack tip as well as loading and support points.

## 7. Conclusions

Presented analytical solutions enable the straightforward determination of the AENF specimen compliance as a function of crack length, specimen geometry, and applied downward force using the Euler–Bernoulli simple beam theory as well as the more refined Timoshenko beam theory, including deformation due to shearing. A very good convergence of results has been obtained comparing deflection profiles of the analytical and numerical modeled AENF beams. In addition, experimental tests including deflection of the central point of the beam have agreed with the theoretical predictions.

The rotation of cross-section during AENF tests influences specimen compliance and increases the accuracy of the theoretical model when compared to the experimental tests. In addition, the inclusion of cross-section rotation with respect to the laminate neutral plane changes the nature of shear force transition from the undelaminated part of the beam (sublaminate 3 and 4) into the cracked parts, which are more uniformly distributed in TBT, especially for relatively short cracks. Performed numerical analyses have proven that the AENF specimen is subjected to the complex compression/tension and bending moment loadings, ahead of the crack tip in the undelaminated part of the beam. Such an advanced loading state changes into pure bending moment loading in the delaminated part of the AENF specimens. The analyses of in-plane shearing forces of symmetric ENF and asymmetric AENF have revealed that the three-point bending test on delaminated beams generates some anti-planar shear contribution, especially in the edge of the crack tip.

## Figures and Tables

**Table 1:** Results of the AENF specimen deflection at mid-span during loading.

Crack Length [mm]	15	30	45	15	30	45	15	30	45	15	30	45
Layup	η = 1			η = 1.85			η = 2.35			η = 3.97		
*δ_EXP_* (mm)	0.515	0.668	1.079	0.554	0.690	1.025	0.518	0.619	0.853	0.519	0.569	0.686
*δ_SBT_* (mm)	0.506	0.643	1.017	0.540	0.647	0.936	0.516	0.597	0.815	0.517	0.562	0.684
*δ_TBT_* (mm)	0.520	0.657	1.031	0.554	0.662	0.952	0.530	0.612	0.831	0.532	0.577	0.700
*δ_FEA_* (mm)	0.532	0.671	1.045	0.569	0.698	0.965	0.547	0.647	0.854	0.550	0.606	0.723

## Appendix A. Sublaminate Equivalent Stiffness

According to Classical Laminated Plate Theory, the asymmetrical laminated beam can be treated as homogeneous when its equivalent stiffnesses are known. A detailed schematization for the calculation of equivalent stiffness according to the laminate coordinates $z_{i}$ is described in [21].

(A1) $$A=\overset{n}{\underset{i=1}{\sum }}E_{x}^{\left(i\right)}\left(z_{i}-z_{i-1}\right)$$

(A2) $$B=\frac{1}{2}\overset{n}{\underset{i=1}{\sum }}E_{x}^{\left(i\right)}\left(z_{i}^{2}-z_{i-1}^{2}\right)$$

(A3) $$C=\frac{5}{6}\overset{n}{\underset{i=1}{\sum }}G_{zx}^{\left(i\right)}\left(z_{i}-z_{i-1}\right)$$

(A4) $$D=\frac{1}{3}\overset{n}{\underset{i=1}{\sum }}E_{x}^{\left(i\right)}\left(z_{i}^{3}-z_{i-1}^{3}\right)$$

The general equations to calculating longitudinal, transversal extension elastic modulus E as well as shearing modulus G, for sublaminates to SBT with arbitrary stacking sequence are described as follows [8]:

(A5) $$E_{\left(x\right)}=\overset{n}{\underset{i=1}{\sum }}\left[E_{\left(x\right)}^{\left(material\;i\right)}\left(z_{i}-z_{i-1}\right)\right]/\left(z_{n}-z_{0}\right)$$

(A6) $$G_{\left(x\right)}=\overset{n}{\underset{i=1}{\sum }}\left[G_{\left(x\right)}^{\left(material\;i\right)}\left(z_{i}-z_{i-1}\right)\right]/\left(z_{n}-z_{0}\right)$$

(A7) $$\nu_{\left(x\right)}=\sum_{i=1}^{n}\left[\nu_{\left(x\right)}^{\left(material\;i\right)}\left(z_{i}-z_{i-1}\right)\right]/\left(z_{n}-z_{0}\right).$$
